# Pharmacodynamics of aztreonam/ceftazidime/avibactam and polymyxin B versus New Delhi MBL-producing Acinetobacter baumannii

**DOI:** 10.1093/jacamr/dlaf068

**Published:** 2025-05-02

**Authors:** Jacob T Dumbleton, Anant P Shah, Brian M Ho, Navaldeep Singh, Harriet de Souza, Nicholas M Smith

**Affiliations:** Department of Pharmacy Practice, School of Pharmacy and Pharmaceutical Sciences, University at Buffalo, The State University of New York, Buffalo, NY, USA; Department of Pharmacy Practice, School of Pharmacy and Pharmaceutical Sciences, University at Buffalo, The State University of New York, Buffalo, NY, USA; Department of Pharmacy Practice, School of Pharmacy and Pharmaceutical Sciences, University at Buffalo, The State University of New York, Buffalo, NY, USA; Department of Pharmacy Practice, School of Pharmacy and Pharmaceutical Sciences, University at Buffalo, The State University of New York, Buffalo, NY, USA; Department of Pharmacy Practice, School of Pharmacy and Pharmaceutical Sciences, University at Buffalo, The State University of New York, Buffalo, NY, USA; Department of Pharmacy Practice, School of Pharmacy and Pharmaceutical Sciences, University at Buffalo, The State University of New York, Buffalo, NY, USA

## Abstract

**Background:**

*Acinetobacter baumannii* has become an increasingly urgent public health concern among global health agencies due to high rates of carbapenem resistance. Carbapenem-resistant *A. baumannii* (CRAB) that express both oxacillinases and MBLs is especially problematic due to resistance to all β-lactams.

**Methods:**

Two clinical *A. baumannii* isolates, AR-0033 and AR-0083, harbouring *bla*_NDM-1_ (for both isolates MIC_aztreonam_ >64 mg/L, MIC_ceftazidime/avibactam_ >128/4 mg/L, MIC_polymyxin B_ = 1 mg/L, MIC_cefiderocol_  ≥16 mg/L) were treated with mono- or combination therapies of aztreonam/ceftazidime/avibactam and polymyxin B (PMB) in static time–kill studies over 24 h. Replicate time–kills were analysed by integrating the area under the cfu/mL-versus-time curve using the linear-trapezoidal method and normalizing to the growth control to produce the log-ratio area (LRA). The LRA was mathematically modelled as a function of aztreonam concentrations using a Hill-type function to identify the IC_50_ values for aztreonam.

**Results:**

Treatment with aztreonam/ceftazidime/avibactam achieved <2 log_10_ cfu/mL reduction by 24 h for all concentrations in both isolates. Monotherapies of PMB at 0.75, 1.5, 3.0 and 6.0 mg/L displayed maximum killing by 6 h against AR-0033. Monte Carlo simulations of human pharmacokinetics of aztreonam showed that package insert dosing resulted in a average free steady-state concentration above the target aztreonam IC_50_ values for AR-0033 ≥96% of time when in combination with ceftazidime/avibactam and PMB.

**Conclusions:**

This study supports the potential utility of low-dose PMB therapy in combination with β-lactams to combat NDM-producing CRAB.

## Introduction

*Acinetobacter baumannii* is a Gram-negative, aerobic, non-fermenting bacillus that frequently causes highly drug-resistant nosocomial infections.^1^  *A. baumannii* is an increasingly urgent concern among global health agencies, with the CDC and WHO declaring carbapenem-resistant *A. baumannii* (CRAB), respectively, as an urgent and critical priority.^2,3^  *A. baumannii* has a natural competency that allows it to readily acquire new antibiotic resistance genes and can survive for extended periods on healthcare facility surfaces, which has led to the need for new treatment options. Infections associated with *A. baumannii* often include bacteraemia, meningitis, pneumonia, and infections of the skin and soft tissues, surgical wounds, or urinary tract.^4,5^ Reported mortality rates of CRAB infections range from 16% to 76%, depending upon site of infection and initial treatment.^2,6^ Despite a reported decrease in total CRAB-associated infections—11 700 were reported by the CDC in 2012 compared with 8500 reported in 2017—there are still areas of the world with carbapenem resistance rates greater than 50%.^3^

MDR and XDR *A. baumannii* has become increasingly problematic due to the presence of class D carbapenemases (i.e. oxacillinase), MBLs or, most problematically, both. This surge in carbapenem resistance in *A. baumannii* has resulted in limited treatment options. NDM-1, an MBL, confers resistance to all β-lactams with the exclusion of aztreonam, whereas oxacillinases confer additional resistance to carbapenems and aztreonam.^7–9^ The antibiotic pipeline for Gram-negative bacteria has largely focused on the development of novel β-lactamase inhibitors, but recently approved inhibitors have no activity against MBLs and only modest activity against oxacillinases.^10,11^

Because of the unique enzyme profile of CRAB, clinicians are often forced to use the polymyxins [polymyxin B and polymyxin E (colistin)] as salvage therapy for CRAB infections, which have a narrow therapeutic window. Polymyxin doses are typically limited by neuro- and nephrotoxicity and therefore have a guideline-recommended maximum daily exposure [i.e. 24 h area under the concentration–time curve at steady state (AUC_ss,24_)] of 100 mg h L^−1^.^12^ The current standard of care for NDM-harbouring Gram-negatives is treatment with either polymyxins or aztreonam-based combinations (typically with ceftazidime/avibactam). However, CRAB is intrinsically resistant to aztreonam, with neither CLSI nor EUCAST providing breakpoints. Thus, combating NDM-harbouring CRAB demands significant innovation to address this public health threat and develop rationally optimized combination therapies.

The main objective of this study was to evaluate the bacterial killing effects of polymyxin B alone and as a low-dose adjuvant to combination aztreonam/ceftazidime/avibactam therapy for the treatment of NDM-expressing, carbapenem-resistant *A. baumannii*. To achieve this goal, we studied humanized concentrations of each antibiotic *in vitro* against two clinical isolates of *A. baumannii* that both expressed NDM-1.

## Methods

### Bacterial isolates, antibiotics and media

Experiments were conducted using two NDM-1–harbouring *A. baumannii* isolates: AR-0033 and AR-0083 (both isolates: MIC_aztreonam_ >64 mg/L, MIC_ceftazidime/avibactam_ >128/4 mg/L, MIC_polymyxin B_ = 1 mg/L, MIC_cefiderocol_ ≥16 mg/L). MICs for both isolates were determined via broth microdilution in duplicate according to CLSI. AR-0033 harbours the β-lactamase genes *bla*_NDM-1_ and *bla*_OXA-94_; comparatively, AR-0083 harbours *bla*_NDM-1_, *bla*_OXA-23_, *bla*_OXA-69_ and *bla*_PER-7_. Avibactam (IMHA, Inc., Mount Prospect, IL, USA), aztreonam (AKSci, Union City, CA, USA), ceftazidime (Sigma Aldrich, St Louis, MO, USA) and polymyxin B (Sigma Aldrich, St Louis, MO, USA) were used for all studies and prepared fresh for each experiment and reconstituted using saline. CAMHB, supplemented with 25 mg/L calcium and 12.5 mg/L magnesium, and Mueller–Hinton agar (MHA) were used for all experiments.

### Static time–kill studies

Static time–kill studies were conducted in CAMHB using mono- and combination therapies of aztreonam, ceftazidime/avibactam and polymyxin B. Monotherapies included a 2-fold aztreonam/ceftazidime/avibactam concentration array from 16.5/18.25/2.475 mg/L to 132/146/19.8 mg/L, and a 2-fold polymyxin B concentration array from 0.75 mg/L to 6.0 mg/L. Combination therapies included the 2-fold aztreonam/ceftazidime/avibactam concentration array plus the 2-fold polymyxin B concentration array. A target starting inoculum of 10^7^ cfu/mL was used for all experiments, with each reaction vessel being incubated at 37°C in a water bath shaker. Samples were collected at 0, 1, 2, 4, 6, 8 and 24 h and plated on drug-free MHA after serial dilutions with saline. Colony counts were enumerated using a Protos 3 automated colony counter (Synbiosis, Cambridge, UK).

### Data analysis

Data for each experiment were summarized by calculating the area under the cfu-versus-time curve (AUCFU) using the linear trapezoidal rule, as previously described.^13^ The AUCFU for each time–kill was then normalized to the growth control and log_10_-transformed to produce the log ratio area (LRA). Observed changes in LRA due to aztreonam concentrations were fit with a Hill-type function to identify maximum effect (*I*_max_), the extent of drug effect ($I_{\mathrm{delta}}=I_{\max}-I_{0}$), where *I*_0_ is defined as the baseline drug effect normalized by the growth control, and drug sensitivity (IC_50_).

### Monte Carlo simulations

Clinically observed concentrations were compared with estimated IC_50_ values by generating pharmacokinetic (PK) profiles of aztreonam in simulated subjects using established PK models from the literature.^14,15^ Aztreonam dosing was based on the package insert and implemented with two loading doses (i.e. 1 g × 1 at 0 h, and 2 g × 1 at 0 h) with three different maintenance regimens (i.e. 500 mg q8h at 8 h, 1 g q8h at 8 h, and 2 g q8h at 8 h) resulting in a maximum simulated dose of 6 g/day. For each tested dose regimen, 1000 simulated concentration–time profiles were generated to calculate the mean percent elapsed time over IC_50_. Subject simulation data and plot generation were performed using the R software suite (v4.3.0; R Foundation, Vienna, Austria). Likewise, PK profiles of polymyxin B were generated using a similar established PK model, while implementing identical, guideline-recommended loading doses of 2 mg/kg of polymyxin B with five different maintenance regimens (i.e. 0.3125, 0.625, 1.25, 2.5 and 5 mg/kg q12h at 12 h) to determine relevant clinical polymyxin B average steady-state concentration (*f*C_ss,avg_) and percentage of simulated subjects above the guideline-recommended AUC_ss,24_.^12^

## Results

### Static time–kill studies

**Figure 1.**
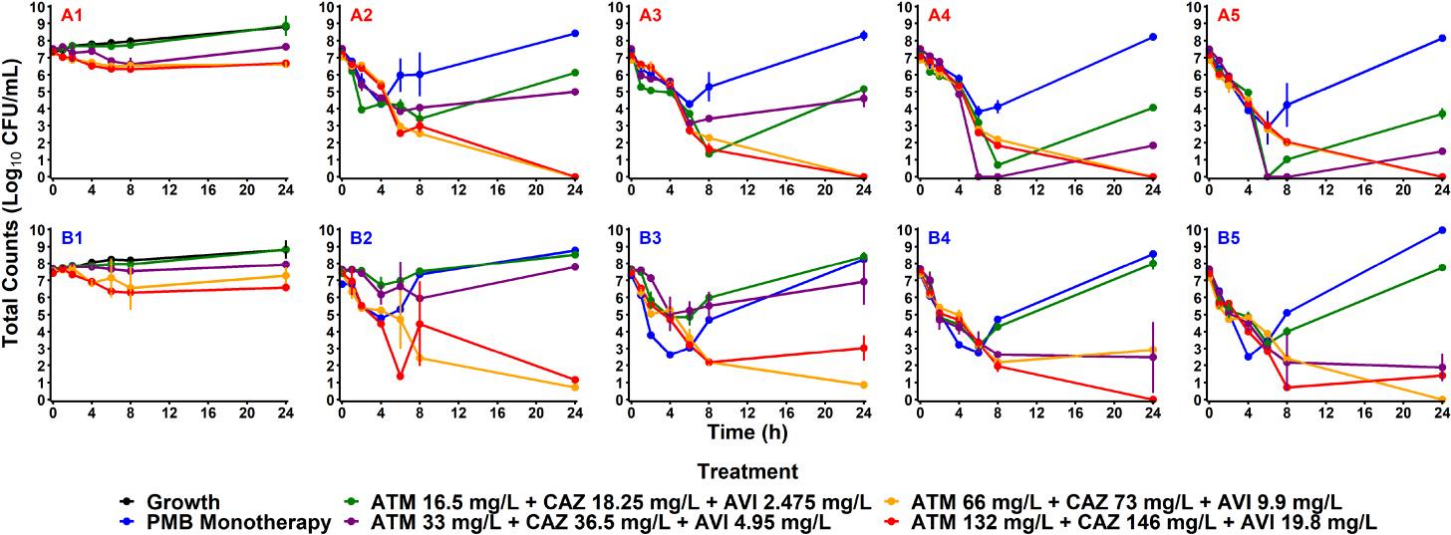
Static time–kill results of *A. baumannii* isolates versus combination β-lactam therapies with and without polymyxin B. Observed bacterial concentrations (cfu/mL) of *A. baumannii* isolates AR-0033 (A1–A5) and AR-0083 (B1–B5) over 24 h as mean (dots) and standard deviation (bars) from replicates. Plots are separated by polymyxin B adjuvant concentrations: polymyxin B 0.0 mg/L (A1/B1), polymyxin B 0.75 mg/L (A2/B2), polymyxin B 1.5 mg/L (A3/B3), polymyxin B 3.0 mg/L (A4/B4) and polymyxin B 6.0 mg/L (A5/B5). ATM, aztreonam; AVI, avibactam; CAZ, ceftazidime; PMB, polymyxin B.

Results of static time–kill studies including aztreonam/ceftazidime/avibactam and polymyxin B monotherapy and combination therapies against both isolates of *A. baumannii* are summarized in Figure 1. Therapies of aztreonam/ceftazidime/avibactam with polymyxin B achieved greater than 2 log_10_ cfu/mL of bacterial reduction by 24 h for all concentrations and isolates. Monotherapies of polymyxin B at 0.75, 1.5, 3.0 and 6.0 mg/L displayed maximum bacterial killing between 4 and 6 h against AR-0033, with maximum reductions of 3.19, 3.17, 3.53 and 4.49 log_10_ cfu/mL, respectively. By comparison, the same polymyxin B concentrations resulted in maximum bacterial killing against AR-0083 between 4 and 8 h, with maximum reductions of 1.68, 2.66, 4.12 and 3.04 log_10_ cfu/mL, respectively. Despite initial bacterial killing, polymyxin B monotherapies resulted in regrowth by 24 h for both isolates.

All of the experimental polymyxin B concentrations when combined with aztreonam/ceftazidime/avibactam at 16.5/18.25/2.475 mg/L or 33/36.5/4.95 mg/L produced bactericidal activity (i.e. 99.9% reduction) against AR-0033, with maximum bacterial killing occurring between 6 and 8 h. Aztreonam/ceftazidime/avibactam concentrations of 16.5/18.25/2.475 mg/L coupled with polymyxin B at 0.75 and 1.5 mg/L achieved log_10_ cfu/mL reductions of 4.04 and 6.02 compared with starting conditions, before regrowing at 24 h. When coupled with polymyxin B at 3.0 and 6.0 mg/L, the treatments achieved transient bactericidal activity before regrowing at 24 h.

In a similar fashion, aztreonam/ceftazidime/avibactam concentrations of 33/36.5/4.95 mg/L coupled with polymyxin B at 0.75 and 1.5 mg/L achieved log_10_ cfu/mL reductions of 3.67 and 4.37, before regrowing at 24 h. When coupled with polymyxin B at 3.0 and 6.0 mg/L, the treatments also displayed bactericidal activity before regrowing at 24 h. All combinations of polymyxin B with aztreonam/ceftazidime/avibactam at 66/73/9.9 mg/L or 132/146/19.8 mg/L achieved bactericidal activity against AR-0033 with no regrowth noted at 24 h.

Against AR-0083, combinations of polymyxin B at any experimental concentration with aztreonam/ceftazidime/avibactam at 16.5/18.25/2.475 mg/L or 33/36.5/4.95 mg/L, achieved maximum bacterial killing between 4 and 8 h. Polymyxin B at 0.75, 1.5, 3.0 and 6.0 mg/L with aztreonam/ceftazidime/avibactam at 16.5/18.25/2.475 mg/L achieved log_10_ cfu/mL reductions of 0.758, 2.81, 4.44 and 4.32, respectively, before regrowing at 24 h. Combinations of aztreonam/ceftazidime/avibactam 33/36.5/4.95 mg/L with polymyxin B at 0.75 and 1.5 mg/L achieved log_10_ cfu/mL reductions of 1.69 and 2.59 before regrowing at 24 h.

Select combinations of polymyxin B and aztreonam/ceftazidime/avibactam at 66/73/9.9 mg/L or 132/146/19.8 mg/L failed to maintain bactericidal activity at 24 h against AR-0083. Combinations of aztreonam/ceftazidime/avibactam at 66/73/9.9 mg/L with polymyxin B at 3.0 mg/L achieved bactericidal activity at approximately 8 h but showed regrowth by 24 h, whereas aztreonam/ceftazidime/avibactam at 132/146/19.8 with polymyxin B at 1.5 and 6.0 mg/L showed bactericidal activity at approximately 8 h but regrew by 24 h.

### Hill-type function

**Figure 2.**
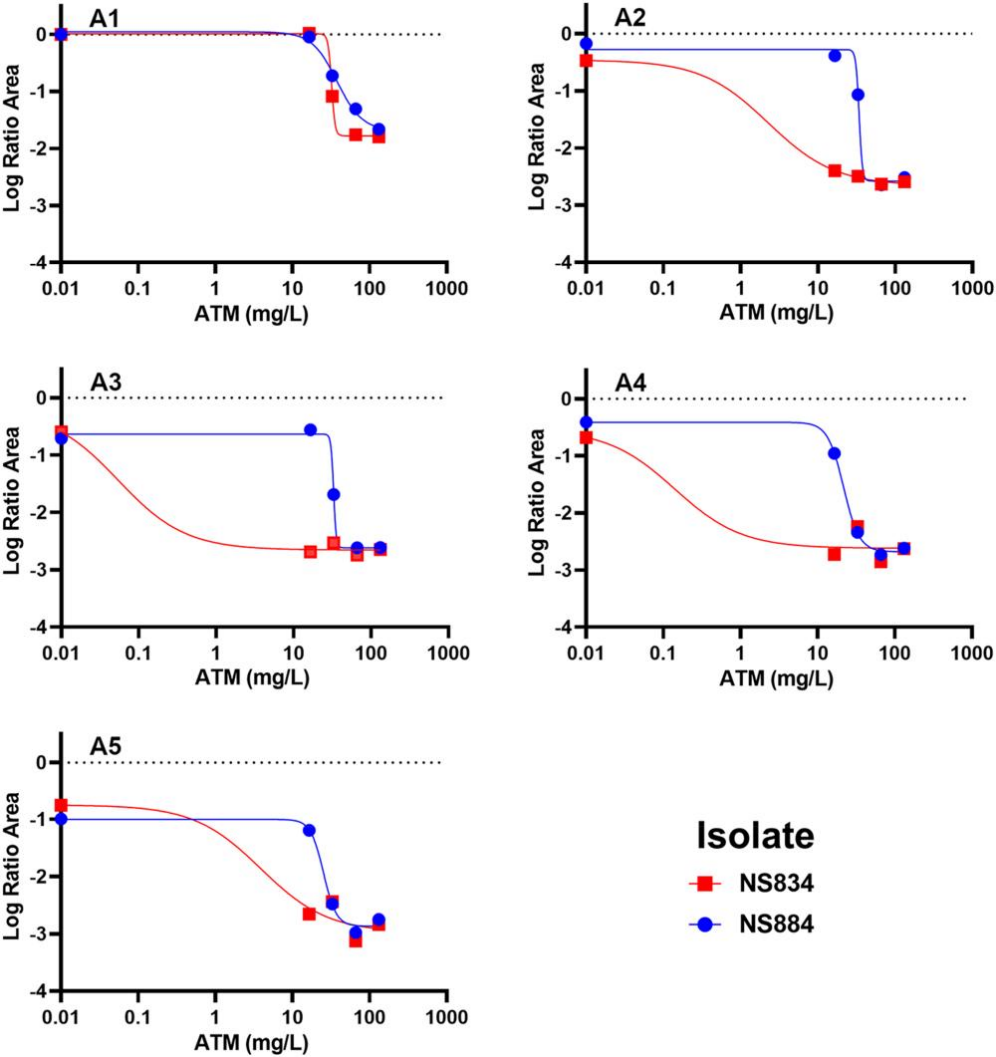
Hill-type function modelled over observed log ratio area. A Hill-type function modelled over recorded changes in growth-normalized log ratio area due to polymyxin B concentrations to determine *I*_max_ and IC_50_. Aztreonam (ATM) concentrations are in the aztreonam/ceftazidime/avibactam combination therapy ratio of 1.1:0.15:1. Plots are separated by polymyxin B adjuvant concentrations: polymyxin B 0.0 mg/L (A1), polymyxin B 0.75 mg/L (A2), polymyxin B 1.5 mg/L (A3), polymyxin B 3.0 mg/L (A4) and polymyxin B 6.0 mg/L (A5).

Data from static time–kill studies were best fit with a Hill-type function and are summarized in Figure 2. Aztreonam/ceftazidime/avibactam concentrations yielded aztreonam IC_50_ values of 32.2 mg/L and 37.9 mg/L against AR-0033 and AR-0083. Upon adding polymyxin B at 0.75, 1.5, 3.0 and 6.0 mg/L to aztreonam/ceftazidime/avibactam combinations against AR-0033, subsequent IC_50_ values were reduced to 2.26, 0.0543, 0.141 and 3.90 mg/L, respectively. Addition of the same polymyxin B array to aztreonam/ceftazidime/avibactam combinations against AR-0083 reduced subsequent IC_50_ values to 34.1, 32.8, 21.7 and 25.3 mg/L, respectively.

### Monte Carlo simulations

**Figure 3.**
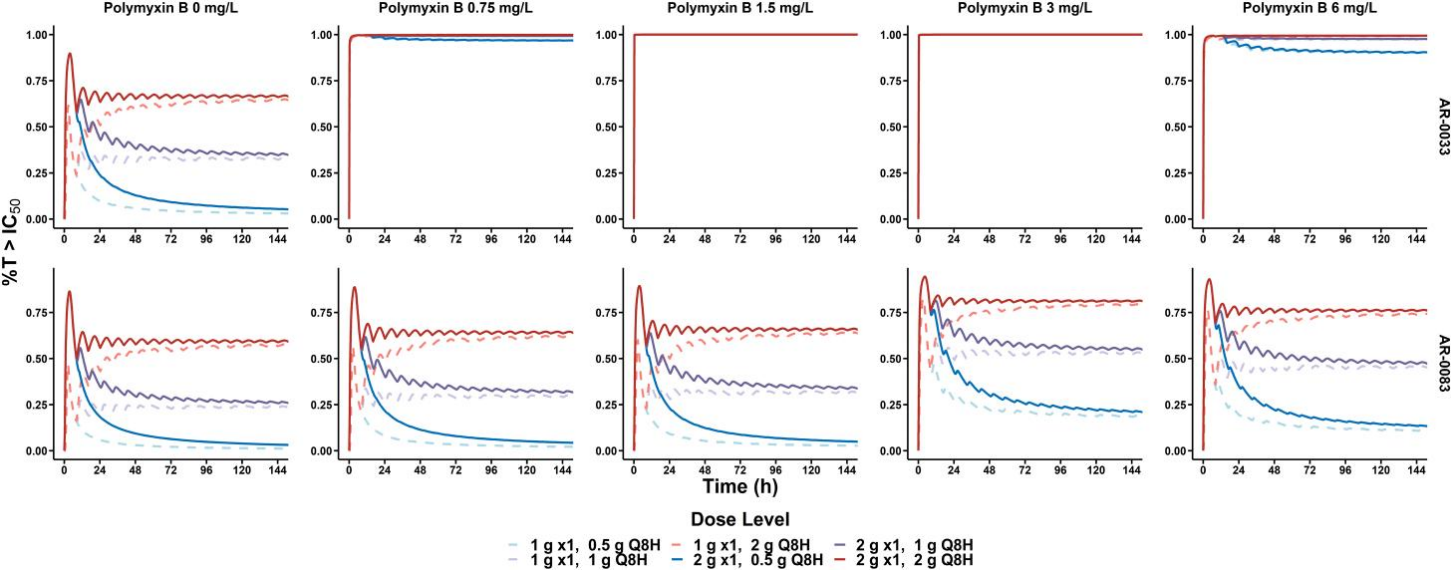
Percentage of time above estimated IC_50_ values of aztreonam. Six simulated dosing regimens over the course of 144 h to determine the percentage of time a simulated subject would have a *f*C_ss,avg_ above the estimated aztreonam IC_50_ value. Aztreonam was simulated as a maintenance dose of 500 mg, 1 g or 2 g every 8 h, with either a 1 g (light-shaded dashed line) or 2 g (dark-shaded solid line) loading dose.

Simulated maintenance dosing regimens of aztreonam resulted in a *f*C_ss,avg_ ± standard deviation (SD) of 5.27 ± 1.30 mg/L for the 500 mg q8h regimen, whereas 1 g q8h and 2 g q8h regimens resulted in *f*C_ss,avg_ of 10.5 ± 2.61 mg/L and 21.1 ± 5.22 mg/L, respectively. No difference in *f*C_ss,avg_ was observed between regimens using a 1 g loading dose, as opposed to a 2 g loading dose. Data from simulated dosing regimens were plotted as a function of percentage of time above the IC_50_ values of aztreonam in Figure 3.

Package insert dosing of aztreonam, using a 2 g loading dose followed by 2 g q8h starting at 8 h resulted in simulated subjects having a mean *f*C_ss,avg_ above the estimated IC_50_ for aztreonam in the presence of ceftazidime/avibactam for 58.9% of time for AR-0083. Addition of the polymyxin B array at 0.75, 1.5, 3.0 and 6.0 mg/L to aztreonam/ceftazidime/avibactam increased the percentage of time above the estimated IC_50_ to 64.5%, 65.4%, 80.9% and 75.7%, respectively. In contrast, simulated subjects with an identical regimen had a median *fC*_ss,avg_ above the estimated IC_50_ for aztreonam in the presence of ceftazidime/avibactam for 66.2% of time for AR-0033. Adding the same polymyxin B array increased the percentage of time to greater than 99% for all concentrations of polymyxin B.

Dosing of aztreonam at a 2 g loading dose, followed by 1 g q8h starting at 8 h resulted in simulated subjects having a mean *f*C_ss,avg_ above the estimated IC_50_ for aztreonam in the presence of ceftazidime/avibactam for 25.9% of time for AR-0083. The percentage of time above the estimated IC_50_ was increased to 31.6%, 33.7%, 54.6% and 47.2% upon addition of the polymyxin B array. Comparatively, simulated subjects with an identical regimen had a mean *fC*_ss,avg_ above the estimated IC_50_ for aztreonam in the presence of ceftazidime/avibactam for 34.7% of time for AR-0033. The addition of the same polymyxin B array increased the percentage of time to greater than 99% for all concentrations of polymyxin B except polymyxin B 6.0 mg/L, which increased to 97.6%.

Aztreonam dosing regimens at a 2 g loading dose, followed by 500 mg q8h starting at 8 h resulted in simulated subjects having a mean *f*C_ss,avg_ above the estimated IC_50_ for aztreonam in the presence of ceftazidime/avibactam for 3.3% of the time for AR-0083. When adding the polymyxin B array, subsequent percentages above the estimated IC_50_ were increased to 4.6%, 5.1%, 21.1% and 13.5%, respectively. In comparison, simulated subjects with an identical regimen had a mean *fC*_ss,avg_ above the estimated IC_50_ for aztreonam in the presence of ceftazidime/avibactam for 5.4% of the time for AR-0033. Upon addition of the polymyxin B array, subsequent percentages above the estimated IC_50_ were increased to 96.8%, 99.9%, 99.9% and 90.2%, respectively.

Regarding polymyxin B, simulations of critically ill subjects using a polymyxin B maintenance regimen of 0.3125 mg/kg produced a median *f*C_ss,avg_ of 0.400 ± 0.141 mg/L. Increasing the polymyxin B maintenance regimens 2-fold to 5 mg/kg produced median *f*C_ss,avg_ values of 0.788 ± 0.271 mg/L, 1.57 ± 0.533 mg/L, 3.13 ± 1.06 mg/L, and 6.26 ± 2.10 mg/L, respectively. The 2.5 mg/kg and 5 mg/kg maintenance regimens had 16.8% and 90.2% of simulated subjects above the guideline-recommended AUC_ss,24_ of 100 mg h L^−1^, whereas the 0.3125, 0.625 and 1.25 mg/kg maintenance regimens had 0% of simulated subjects above the guideline-recommended AUC_ss,24_.^12^

## Discussion

The rise and propagation of MDR and XDR *A. baumannii* in clinical settings has prompted the need for new combination therapies. Combinations of ceftazidime/avibactam plus aztreonam have been successful against NDM-1–harbouring Enterobacterales.^16,17^ Yet, when this combination triple therapy was used against *A. baumannii* isolates AR-0033 and AR-0083 in this study, all combinations of aztreonam/ceftazidime/avibactam failed to produce at least a 2 log_10_ cfu/mL bacterial killing, further indicating the need for alternative strategies against MBL-harbouring *A. baumannii*.

Treatment with polymyxins is a last-line option against CRAB, but this strategy is limited by dose-dependent toxicities and resistance development.^18^ To combat this, polymyxin B was tested as a low-, normal- and high-concentration adjuvant administered alongside aztreonam/ceftazidime/avibactam. Aztreonam/ceftazidime/avibactam and polymyxin B combination therapy resulted in bactericidal activity against AR-0033. When this procedure was repeated against AR-0083, therapeutic and supratherapeutic concentrations of aztreonam/ceftazidime/avibactam combinations yielded minor additional bacterial killing when compared with polymyxin B monotherapy.

The mechanisms of polymyxin B synergy on β-lactams appear multifactorial, related to the direct killing effects of each drug, cross-coverage of resistant subpopulations, and mechanistic synergy. Regarding mechanistic synergy, it is hypothesized that polymyxin B acts with detergent-like properties at the outer membrane, leading to increased β-lactam target site concentrations through enhanced β-lactam permeability.^19^ Potential limitations of aztreonam/ceftazidime/avibactam therapy in this study may be due to the common β-lactamases expressed in *A. baumannii* compared with the Enterobacterales. Specifically, *A. baumannii* AR-0083 harbours *bla*_PER-7_ and *bla*_OXA-23_, which have been shown to produce rapid hydrolysis of aztreonam, unlike *bla*_OXA-94_ of AR-0033, which degrades aztreonam more slowly.^20^ This is supported by the initial reduction of approximately 93% in aztreonam drug IC_50_ when polymyxin B 0.75 mg/L was added to aztreonam/ceftazidime/avibactam against AR-0033, whereas AR-0083 only had an approximate 9% reduction in aztreonam IC_50_.

Simulated clinical trials of aztreonam therapy provide additional insights into estimating the probability of target attainment by allowing the prediction of the *in vivo* response with only *in vitro* observations.^14,15^ Simulated package insert regimens of aztreonam for subjects with severe systemic or life-threatening infections showed that simulated subjects receiving both aztreonam and polymyxin B consistently exceeded the baseline percent time over IC_50_ target of aztreonam monotherapy for both strains. Simulated AR-0033 subjects exceeded the observed aztreonam IC_50_ efficacy targets over 99% of time at the maximum simulated dose of 2 g q8h. Meanwhile, simulated AR-0083 subjects in an identical regimen exceeded the observed aztreonam efficacy targets for at least 64% of time.

Simulated guideline dosing of polymyxin B resulted in no simulated subjects exceeding guideline maximum exposure of 100 mg h L^−1^. Only among supratherapeutic maintenance regimens did simulated subjects exceed the guideline maximum toxicity threshold, with the 2.5 mg/kg and 5 mg/kg regimens resulting in 16.8% and 90.2% of simulated subjects being above the guideline maximum toxicity threshold. These data support the use of previously reported de-escalation strategies for polymyxin B in cases of combination use, which can result in human exposures below guideline-based limits of dosing.^12,21^

This study is principally limited by the number of isolates studied, variety of MBLs studied and use of static concentrations. As the studies were conducted at static concentrations, no information regarding drug effects under dynamic concentrations was generated. Future studies could address this through exploration of treatment regimens in a dynamic chemostat model, hollow-fibre infection model or other *in vivo* experimentation methods. In summary, low-dose concentrations of polymyxin B were synergistic with aztreonam/ceftazidime/avibactam concentrations that reflected clinical *fC*_max_ and produced bactericidal activity against both AR-0033 and AR-0083. Ultimately, further research is needed to develop therapies against the growing threat of *A. baumannii* co-expressing MBL and OXA.

## References

[1] Howard A, O'Donoghue M, Feeney A et al *Acinetobacter baumannii*: an emerging opportunistic pathogen. Virulence 2012; 3: 243–50. 10.4161/viru.19700 22546906 PMC3442836

[2] CDC . Antibiotic resistance threats in the United States, 2019. US Department of Health and Human Services, 2019. https://www.cdc.gov/antimicrobial-resistance/data-research/threats/index.html

[3] World Health Organization . Prioritization of pathogens to guide discovery, research and development of new antibiotics for drug-resistant bacterial infections, including tuberculosis. 2017. https://www.who.int/publications/i/item/WHO-EMP-IAU-2017.12

[4] Khaledi M, Shahini Shams Abadi M, Validi M et al Phenotypic and genotypic detection of metallo-beta-lactamases in *A. baumanii* isolates obtained from clinical samples in Shahrekord, southwest Iran. BMC Res Notes 2019; 12: 597. 10.1186/s13104-019-4636-y 31533853 PMC6751628

[5] Alkasaby NM, El Sayed Zaki M. Molecular study of *Acinetobacter baumannii* isolates for metallo-β-lactamases and extended-spectrum-β-lactamases genes in intensive care unit, Mansoura University Hospital, Egypt. Int J Microbiol 2017; 2017: 3925868. 10.1155/2017/3925868 28567057 PMC5439075

[6] Lemos EV, de la Hoz FP, Einarson TR et al Carbapenem resistance and mortality in patients with *Acinetobacter baumannii* infection: systematic review and meta-analysis. Clin Microbiol Infect 2014; 20: 416–23. 10.1111/1469-0691.12363 24131374

[7] Da Silva GJ, Domingues S. Insights on the horizontal gene transfer of carbapenemase determinants in the opportunistic pathogen *Acinetobacter baumannii*. Microorganisms 2016; 4: 29. 10.3390/microorganisms4030029 27681923 PMC5039589

[8] Kaur A, Gupta V, Chhina D. Prevalence of metallo-β-lactamase-producing (MBL) *Acinetobacter* species in a tertiary care hospital. Iran J Microbiol 2014; 6: 22–5. 25954487 PMC4419041

[9] Nordmann P, Poirel L, Toleman MA et al Does broad-spectrum beta-lactam resistance due to NDM-1 herald the end of the antibiotic era for treatment of infections caused by Gram-negative bacteria? J Antimicrob Chemother 2011; 66: 689–92. 10.1093/jac/dkq520 21393184

[10] Lomovskaya O, Sun D, Rubio-Aparicio D et al Vaborbactam: spectrum of beta-lactamase inhibition and impact of resistance mechanisms on activity in Enterobacteriaceae. Antimicrob Agents Chemother 2017; 61: e01443-17. 10.1128/AAC.01443-17 28848018 PMC5655098

[11] Livermore DM, Warner M, Mushtaq S. Activity of MK-7655 combined with imipenem against Enterobacteriaceae and *Pseudomonas aeruginosa*. J Antimicrob Chemother 2013; 68: 2286–90. 10.1093/jac/dkt178 23696619

[12] Tsuji BT, Pogue JM, Zavascki AP et al International consensus guidelines for the optimal use of the polymyxins: endorsed by the American College of Clinical Pharmacy (ACCP), European Society of Clinical Microbiology and Infectious Diseases (ESCMID), Infectious Diseases Society of America (IDSA), International Society for Anti-infective Pharmacology (ISAP), Society of Critical Care Medicine (SCCM), and Society of Infectious Diseases Pharmacists (SIDP). Pharmacotherapy 2019; 39: 10–39. 10.1002/phar.2209 30710469 PMC7437259

[13] Lenhard JR, von Eiff C, Hong IS et al Evolution of *Staphylococcus aureus* under vancomycin selective pressure: the role of the small-colony variant phenotype. Antimicrob Agents Chemother 2015; 59: 1347–51. 10.1128/AAC.04508-14 25451045 PMC4335873

[14] Sandri AM, Landersdorfer CB, Jacob J et al Population pharmacokinetics of intravenous polymyxin B in critically ill patients: implications for selection of dosage regimens. Clin Infect Dis 2013; 57: 524–31. 10.1093/cid/cit334 23697744

[15] Xu H, Zhou W, Zhou D et al Evaluation of aztreonam dosing regimens in patients with normal and impaired renal function: a population pharmacokinetic modeling and Monte Carlo simulation analysis. J Clin Pharmacol 2017; 57: 336–44. 10.1002/jcph.810 27530649

[16] Marshall S, Hujer AM, Rojas LJ et al Can ceftazidime-avibactam and aztreonam overcome β-lactam resistance conferred by metallo-β-lactamases in Enterobacteriaceae? Antimicrob Agents Chemother 2017; 61: e02243-16. 10.1128/AAC.02243-16 28167541 PMC5365724

[17] Lodise TP, Smith NM, O'Donnell N et al Determining the optimal dosing of a novel combination regimen of ceftazidime/avibactam with aztreonam against NDM-1-producing Enterobacteriaceae using a hollow-fibre infection model. J Antimicrob Chemother 2020; 75: 2622–32. 10.1093/jac/dkaa197 32464664 PMC8444334

[18] Smith NM, Lenhard JR, Boissonneault KR et al Using machine learning to optimize antibiotic combinations: dosing strategies for meropenem and polymyxin B against carbapenem-resistant *Acinetobacter baumannii*. Clin Microbiol Infect 2020; 26: 1207–13. 10.1016/j.cmi.2020.02.004 32061797 PMC7587610

[19] Mohapatra SS, Dwibedy SK, Padhy I. Polymyxins, the last-resort antibiotics: mode of action, resistance emergence, and potential solutions. J Biosci 2021; 46: 85. 10.1007/s12038-021-00209-8 34475315 PMC8387214

[20] Evans BA, Amyes SG. OXA β-lactamases. Clin Microbiol Rev 2014; 27: 241–63. 10.1128/CMR.00117-13 24696435 PMC3993105

[21] Smith NM, Boissonneault KR, Chen L et al Mechanistic insights to combating NDM- and CTX-M-coproducing *Klebsiella pneumoniae* by targeting cell wall synthesis and outer membrane integrity. Antimicrob Agents Chemother 2022; 66: e0052722. 10.1128/aac.00527-22 35924913 PMC9487485

